# On the Danger of Using Certain Washes in Ulceration of the Cornea

**Published:** 1846-04

**Authors:** 


6. "*On the danger of using certain washes in cases of Ulceration of the Cornea.* By Dr. Cunier.—We have already directed the attention of practitioners to this matter, by pointing out the serious consequences which may ensue from indulging in the common practice of uniting laudanum with the metallic salts of lead, silver, zinc, copper, cadmium, &c., in preparing collyria. From such combination, says the skilful opthalmologist of Brussels, an insoluble meconate is precipitated, which, by the shaking of the wash when it is about to be used, may be brought into contact with the eye, and become incrusted in the ulceration of the cornea. The result of some recent experiments instituted by Dr. Cunier, at the request of M. Boyer, established beyond doubt the fact, that this decomposition does in reality take place, and that accidents likely to arise from it are such as were previously indicated by the experiments. That, in fact, the precipitate formed by the addition of laudanum to washes made with the sulphates of zinc, copper and cadmium, is less abundant than in those made with the acetate of lead or nitrate of silver; nevertheless, in the quantity in which it is produced, it suffices to cause incrustations in the membrane, when used for ulcerations of the cornea.

"In concluding his article, Dr. Cunier indicates a very simple means for avoiding this difficulty. It consists in taking the precaution, when ordering these collyria, to combine the metallic salt with a salt of morphia *of the same acid;* for instance, the acetate of morphia with the metallic acetates; the sulphate of morphia with the metallic sulphates, &c., &c. In this way the same clinical result is obtained, without the patient being exposed to the serious consequences which may arise from incrustations."—*Gazette Médicale de Paris*, in *Ibid.*

